# Anti-inflammatory and biologic drugs for atopic dermatitis: a therapeutic approach in children and adolescents

**DOI:** 10.3389/fmed.2023.1214963

**Published:** 2023-08-16

**Authors:** Carlo Caffarelli, Arianna Giannetti, Giuliana Giannì, Giampaolo Ricci

**Affiliations:** ^1^Clinica Pediatrica, Azienda Ospedaliero-Universitaria, Department of Medicine and Surgery, Università di Parma, Parma, Italy; ^2^Paediatrics Unit, IRCCS Azienda Ospedaliero-Universitaria di Bologna, Bologna, Italy; ^3^Department of Medical and Surgical Sciences (DIMEC), University of Bologna, Bologna, Italy

**Keywords:** atopic dermatitis, biologics, small molecules, dupilumab, tralokinumab, lebrikizumab, Janus Kinase, children

## Abstract

Atopic dermatitis (AD) is a chronic inflammatory disease with a heterogeneous pathogenesis correlated with dysregulation of the immune system and a prevalence of the T2-mediated immune pathway. Recent understanding of the pathogenesis of AD has allowed the development of new drugs targeting different mechanisms and cytokines that have changed the treatment approach. The aim of this review is to update knowledge on the standard of care and recent advancements in the control of skin inflammation. In light of recent guidelines, we report on the clinical efficacy of novel treatments, with special attention to situations where biologics and small molecules are involved.

## Introduction

Atopic dermatitis (AD) is a chronic relapsing inflammatory skin disease associated with dysregulation of the immune system and Th2 immune responses (1). It represents the most frequent skin disease in childhood. The multidisciplinary approach includes complex pharmacological treatments, management of allergies (2–4), and behavioral and psychological problems (5).

Treatment is based on the severity of AD scores such as the Severity ScoRing of Atopic Dermatitis (SCORAD) index (6), the Investigator’s Global Assessment (IGA) score (7), or the Eczema Area and Severity Index (EASI) (8). Regular use of emollients with any medication is the cornerstone of AD management. Most of the newer drugs will not be sufficient if emollients are not applied. However, they were not included in this review because our aim was to focus on the most commonly used and novel medications for pediatric AD skin inflammation in light of current recommendations and recent advances.

The introduction of new biologic drugs, such as dupilumab, has significantly transformed the therapeutic landscape for patients with AD. These biologics target specific molecules and pathways that are involved in the inflammatory process, providing a more precise and effective treatment approach for moderate-to-severe AD (Table 1).

**TABLE 1:** Mechanisms of action of drugs used in AD.

Drug	Mechanism of action
**Non-biologic drugs**	
Corticosteroids	Non-genomic, local vasoconstriction (topical formulation); genomic, regulatory effect at the cellular level by inhibiting the transcription of pro-inflammatory cytokines, stimulating the expression of genes encoding anti-inflammatory cytokines, and indirectly regulating transcription by blocking the other transcription factors.
Calcineurin inhibitors	Inhibition of phosphatase activity of calcineurin, which leads to decreased transcription of several T2 cytokines, expression downregulation of the high-affinity receptor for immunoglobulin E (FcεRI) on Langerhans cells, suppression of T cell activation and proliferation, inhibition of the activation of sensory nerves, reduction of local S. aureus colonization and increase of microbial diversity.
Anti-H1 antihistamines	Inverse antagonists of H1 receptors on sensory (pruritus) and cerebral (sedation) nerves
Allergen-specific immunotherapy	Regulation of allergic responses by activation of regulatory T (Treg) cells, which suppress Th2 cells and cytokines, basophils, eosinophils, and IgE production, Breg cells, regulatory natural killer cells, and regulatory innate lymphoid cells. IgG4 levels are increased.
Cyclosporin A	The binding of cytoplasmic cyclophilins to this complex inhibits calcineurin, which in turn inhibits T cell activation and proliferation.
Methotrexate	Antimetabolite that blocks the synthesis of folate and inhibits the Janus kinase (JAK)/STAT pathway, likely inhibiting T lymphocyte function.
Azathioprine	A purine analog that is converted to the antimetabolite 6-mercaptopurine, which has immunosuppressive activity by inhibiting DNA production, thereby reducing the proliferation of T and B cells.
Mycophenolate mofetil	Inhibition of inosine monophosphate dehydrogenase, which blocks the synthesis of guanosine nucleotides and reduces the proliferation of T and B lymphocytes.
Crisaborole, Difamilast, Roflumilast	Phosphodiesterase-4 inhibitors that increase cAMP levels and reduce the release of proinflammatory cytokines and chemokines from activated T cells and B cells.
Ruxolitinib, Tofacitinib, Brepocitinib, Upadacitinib, Abrocitinib, Baricitinib	JAK inhibitors. They inhibit gene transcription of T1 and T2 cytokines.
Tapinarof	Aryl hydrocarbon receptor antagonist. It inhibits IL-17 production from Th17, increases the activity of cutaneous barrier genes such as filaggrin, and exerts antioxidant activity.
Asivatrep	Transient receptor potential vanilloid subfamily V member 1 (TRPV1) antagonist. Activated TRPV1 on keratinocytes, mast cells, and cutaneous sensory nerves induces the release of IL-31, which mediates itch, and molecules that suppress the Th2 pathway in AD.
**Biologic drugs**	
Dupilumab	Fully humanized monoclonal antibody to the alpha subunit of the IL-4 receptor (IL-4Rα), which blocks the formation of the IL-13 Rα 1/IL-4Rα heterodimer receptor complex with subsequent signaling of IL-4 and IL-13 for T2 activation. Neuronal IL-4Rα and Janus kinase 1 (JAK1) signaling in sensory nerves are involved in itching. IL-4Rα may be more involved in the humoral immune response with IgE production.
Tralokinumab, Lebrikizumab	Anti-IL-13 monoclonal antibodies block the interaction between IL-13 and the receptor, inhibiting the formation of the IL-13Rα1/IL-4Rα complex. IL-13 recruits inflammatory cells, downregulates filaggrin expression, and regulates smooth muscle contraction and mucus production in the airways. The IL-13Rα1 receptor may be more involved in skin inflammation.
Nemolizumab	Anti-IL-31 receptor alpha chain humanized IgG2κ monoclonal antibody that inhibits pruritus mediated by sensory neurons.
Tezepelumab	Anti-thymic stromal lymphopoietin (TSLP) monoclonal antibody, inhibits Th2 cell activation and production and reduces keratinocyte differentiation in skin barrier disorders.
Fezakinumab	Anti-IL-22 monoclonal antibody, which downregulates Th2, Th22, Th17, and Th1.
GBR 830 Rocatinlimab	Anti-OX40 monoclonal antibody that inhibits OX40–OX40 ligand binding with reduced T cell responses and improved Treg cell function.
Omalizumab	Anti-IgE monoclonal antibody that blocks IgE binding with FcεRI.
Mepolizumab	Anti-IL-5 monoclonal antibody inhibiting eosinophil activation and chemotaxis.
Ustekinumab	Anti-IL-12/23p40 monoclonal antibody that blocks receptor binding of inflammatory cytokines on lymphocytes.
Risankizumab	Anti-IL-23p19 monoclonal antibody
Secukinumab	Anti-IL-17A monoclonal antibody blocks receptor binding on keratinocytes and the inflammatory response.
Itepekimab	Anti-IL-33 monoclonal antibody inhibits a pathway causing T2 and non–T2 inflammation.

Clinical trials have demonstrated the remarkable efficacy and safety of dupilumab, leading to its approval for the treatment of moderate to severe atopic dermatitis (AD) in children aged over 6 months of age. Other biologics, such as tralokinumab and lebrikizumab, have also been approved for use in adults and adolescents 12 years of age and older.

Ongoing investigations are exploring the potential of biologics targeting different cytokines and pathways implicated in the pathogenesis of AD. Clinical trials are specifically assessing the efficacy and safety of biologics that target IL-13 alone or in combination with other cytokines (Table 1). These novel agents offer potential additional treatment options for patients who do not respond adequately to existing therapies or who experience side effects (9). It is important to note, however, that these biologics are still in the clinical development phase and have not yet received regulatory approval. Limited pediatric trials have been conducted for these novel biologics, with nemolizumab being studied in adolescents (10).

In addition to biologics, small molecules are also being explored as treatment options for AD. In the context of childhood AD, inhibitors of phosphodiesterase-4 (PDE-4) and Janus Kinase (JAK) have been approved for use (Table 1).

The availability of biologic drugs has expanded the treatment options for patients with moderate-to-severe AD, particularly for those who have not achieved satisfactory results with conventional therapies or who have contraindications to systemic immunosuppressive agents. Biologics provide a more targeted and personalized approach, reducing symptoms, improving quality of life, and potentially preventing long-term complications associated with uncontrolled AD.

It is important to acknowledge that biologics have limitations. They can be costly, require regular administration through injection or infusion, and may have associated side effects. Long-term safety data are still being evaluated, and their use in specific patient populations, such as pregnant women and children, requires further investigation.

In this review, we aim to provide a comprehensive analysis of the clinical efficacy of new treatments for AD.

## Materials and methods

We conducted a literature review using two electronic databases, MEDLINE (PubMed) and the Cochrane Library, to gather information on the use of anti-inflammatory and biological drugs for atopic dermatitis in children and adolescents. The search was conducted by filtering for articles published in the last 5 years (January 2017–March 2023). Only articles written in English were selected. Additional relevant articles known to the authors or identified in the references of the already selected articles were added.

## Medications for AD management

**Topical corticosteroids** (TCSs) are a first-line option for AD (Table 1) (11–14). TCSs with low-to-moderate potency are used in mild AD, with more potency in moderate-to-severe cases (11, 12). Indeed, the majority of patients with mild-to-moderate AD are aged 0–4 years, while those with severe disease are older (Table 2) (15). Early use of adequate-potency TCSs, at the onset of the acute AD flare increases control of inflammation, regenerates the skin barrier, and reduces TCS consumption. Creams are indicated for acute or subacute lesions, ointments for chronic lesions (e.g., lichenified and xerotic lesions), and thick corneal layers (e.g., palmar/plantar regions) (16). The Fingertip Unit defines the right amount of TCS to be applied (17). The Fingertip Unit can be used to reduce parental resistance to TCSs (corticophobia). A moderate-to-high-potency TCS is applied in the acute phase, and a low-to-medium-potency is used as maintenance. In areas with higher absorption (eyelids, genitals, face, and skin folds), low-to-moderate-potency TCSs are given. Bone mineral density in children is not decreased by TCSs (18). Proactive TCS two or three times a week prevents flare relapse (19). Corticophobia has an incidence of up to 60–73% in children with AD or their parents (20). It represent a main cause of non-adherence. Healthcare professionals should prevent cortico phobia by clearly addressing the questions and fears of parents. Adequate information is often not provided (20). Wet-wrap therapy for 2–14 days is a second-line treatment with anti-inflammatory and cooling action in patients >6 months of age (21–23). Following a 5′–15′ warm bath, the skin is dried with the application of diluted (5–10%) TCSs to the skin or an internal dressing (21–23). Bandages are applied for 3–24 h, with the best results during the first week (24). Daytime dressings are not often accepted. Transiently increased cortisol levels or infections may occur.

**TABLE 2:** Patient characteristics from a swedish study modified from Ortsater et al. (15).

	Number of subjects, 0–4 years	Number of subjects, 5–9 years	Number of subjects, 10–14 years
Total number of AD patients	65,748	20,305	13,277
Pediatric mild-to-moderate cohort	90%	86%	83%
Pediatric severe cohort	10%	14%	17%

**Topical calcineurin inhibitors (TCIs)** include tacrolimus (tTAC) 0.03% ointment and pimecrolimus (tPIM) 1% cream, which are both approved for AD in patients ≥2 years of age, while tTAC 0.1% is approved for those ≥16 years of age (Table 1) (25–29). TCIs quickly relieve itching and signs with sustained efficacy (30–36). tTAC treatment 2–3 times a week for up to 1 year minimizes TCS consumption and increases the number of days without acute lesions (37, 38). Both tTAC formulations are more effective than low-potency TCSs and comparable to medium-potency TCSs (12, 39). Methylprednisolone 0.1% was significantly better than tTAC 0.03% in reducing EASI, pruritus, insomnia, and costs in children (40). tTAC has greater efficacy than tPIM in children (31, 41). Local burning, prickling, itching, and erythema have been reported, especially in the first days. They are aggravated by sweating. To avoid stinging, TCSs are applied first, followed by tTAC 0.03% and then tTAC 0.1% if possible (12). Allergic contact dermatitis, rosacea-like granulomatous reactions, melanosis of the lips, and viral infections have been reported during TCI treatment (39). Pediatric studies showed a lack of systemic immunosuppression by TCIs over 5 years for tPIM (42) and 10 years for tTAC (36, 43). In 2005, the Food and Drug Administration (FDA) issued a “Black Box warning” regarding the theoretical risk of skin cancer and/or lymphoma associated with TCIs. To date, there is no evidence to support such a risk (44–46). UV protection is still recommended because the risk of photo carcinogenicity increases with long-term use of cyclosporine, a calcineurin inhibitor (47). Experts conclude that tPIM is a safe and effective “steroid-sparing” treatment (39) and that TCIs should no longer be avoided in children >3 months of age (48). Overall, TCIs are a second-line option or an alternative first option for long-term treatment in areas with elevated TCS absorption and atrophy (12, 39). tPIM is suggested for mild AD and tTAC for moderate-to-severe AD and long-term treatment.

**Oral corticosteroids** (Table 1) are a rescue therapy for flares or severe disease (methylprednisolone 1 mg/kg/day for 1–2 weeks). Continuous treatment leads to suppression of the hypothalamic-pituitary-adrenal axis, immunosuppression, hypertension, weight gain, osteoporosis, and growth failure in children (49, 50). Tapering is not required for use for less than 3 weeks; otherwise, the drug should be tapered in approximately 1 month (12, 51).

**Anti-H1 antihistamines** play a controversial role in AD, as itching is not usually linked to histamine. The evidence supporting oral anti-H1 in AD is unclear (52). However, they are included in AD guidelines (51, 53). Sedating first-generation anti-H1s is suggested when itching affects sleep (Table 1) (50, 54, 55). In 2015, the European Medicines Agency (EMA) issued a safety warning on first-generation anti-H1s in children under 2 years of age due to a low risk of QT prolongation and torsades de pointes. A Cochrane review (56) of 25 trials, comprising eight on children, showed no consistent efficacy of second-generation anti-H1s as an “add-on” therapy to topical treatment. Cetirizine and loratadine were not superior to placebo (57). The most common adverse events are sedation (even with non-sedating antihistamines) and cholinergic symptoms (58). First-generation anti-H1s may induce daytime somnolence, impairing school performance and driving (57, 59, 60). Topical antihistamines (e.g., diphenhydramine) are not recommended because of the risk of absorption with systemic toxicity and allergic contact dermatitis (61, 62).

**Allergen-specific immunotherapy** exerts an anti-inflammatory action since it inhibits the response to sensitizing allergens (Table 1). Trials in children with aeroallergen allergies have generally reported its efficacy. However, it should be prescribed to selected children with symptoms following exposure to the relevant allergen (53). Large, controlled studies are warranted for routine use (63, 64).

## Immunosuppressive agents

**Cyclosporin A** (CsA), a member of the calcineurin inhibitor family (65, 66) (Table 1), is approved by the EMA for severe AD in adults and, in some countries, in patients >16 years of age. However, it is widely used in children (67, 68). When other therapies are unavailable or contraindicated, CsA is a first-line option with rapid action and a low incidence of side effects (69). European guidelines endorse CsA with a SCORAD index >50 or persistent AD (62). A dose of 2.5–5 mg/kg/day in two doses is recommended for children and adults (53). In adults (70), higher doses (5 mg/kg per day) achieve a quicker response. CsA and dupilumab proved to be more effective than methotrexate (MTX) and azathioprine (AZA) in terms of severity scores at week 16 (71). AD was significantly more reduced by CsA than by dupilumab at 1 month (72), with no difference between treatments after 4 months (73). CsA is more effective than prednisolone, UVA, and UVB (53). In children aged 2 to 16 years with severe AD, 5 mg/kg/day of CsA was effective either continuously for up to 12 months or in intermittent 12-week courses (74). Consequently, personalized dosing is an option (75). Treatment should not exceed 2 years of continuous regimen (62). Infections, nephrotoxicity, hypertension, tremor, hypertrichosis, headache, gingival hyperplasia, skin cancer, and lymphoma may develop. Nephrotoxicity is more likely when the dose is >5 mg/kg/day. Blood counts and hepatic and renal parameters, in addition to blood pressure, should be monitored (e.g., at baseline, every 4 weeks, and then every 3 months) (62, 70).

**Methotrexate** (MTX) (76–83), **azathioprine** (AZA), and **mycophenolate mofetil** (MMF) (Table 1), which are not approved for AD (70, 84–86), are second-line treatments in severe AD.

A paucity of data shows moderate efficacy and safety of MTX in children and adults (53, 77, 78). MTX and CsA similarly reduced SCORAD in children (79). In adults, CsA and dupilumab showed greater efficacy than MTX (71). Hepatic toxicity, pancytopenia, teratogenicity, and idiopathic pulmonary fibrosis are rare adverse events (80). Blood counts and renal and liver profiles should be monitored (81). The type III procollagen peptide should be checked if available. Folic acid supplementation (5 mg twice weekly) is useful (53, 82, 83). The response to MTX is slow (8–12 weeks) and is maintained over time. The dose is 0.2–0.5 mg/kg/week (maximum 25 mg/week) for 10–16 weeks in children. It is tapered by 2.5–5 mg/week to the lowest effective dose (39).

Azathioprine is less effective than CsA but comparable to MTX (71). The WHO stated that the side effects of AZA outweigh its benefits (86). Improvements occur within 2–3 months with an indeterminate duration (87, 88). Serum thiopurine S-methyltransferase activity allows for safer use of AZA in children (88, 89). When this is unavailable, half the standard dose (2–3 mg/kg/day) for 4–6 weeks is followed by a full dose (53, 70, 84). AZA has hepatotoxicity, myelotoxicity, and carcinogenicity. Blood counts and liver and kidney function must be checked twice monthly for 2 months, then monthly for 4 months, and then every 2 months with an increasing dose (53, 70).

Case reports and open-label studies have reported the benefits of MMF in children and adults with severe AD (90). Improvements occurred within 6–7 weeks. MMF was safer than AZA. Blood counts and renal and liver profiles should be monitored (53). The dose in children is 30–50 mg/kg/day in two doses (91).

## Biologics

Atopic dermatitis is considered a type 2 (T2) disease due to the upregulation of cytokines produced by Th2 lymphocytes and T2 innate lymphoid cells (92). A role for IL-17 and IL-22 is conceivable. Even if the molecular pathophysiology is not totally understood, progress has opened the way to monoclonal antibodies targeting T2 cytokines in moderate-to-severe AD not controlled by TCSs. To date, dupilumab, tralokinumab, and lebrikizumab are marketed for adults and adolescents >12 years of age; dupilumab has been approved by the U.S. Food and Drug Administration and the European Commission for children aged >6 months of age.

**Dupilumab** (93–95) (Table 1) achieved IGA (0, 1), ≥75% of EASI (EASI-75), and reduced SCORAD, pruritus NRS, sleep disturbance, DLQI, and patient-oriented eczema measure (POEM) in several phase III studies in adults with AD for up to 52 weeks (96–98), even when unresponsive to CsA (99–101), and in children aged 6 months to 17 years (102–106). Laboratory monitoring was unnecessary (96–108). However, the rate of conjunctivitis was higher in the dupilumab groups than in the placebo groups at all ages (104, 106, 109). The mechanism remains elusive. Conjunctivitis is treated with corticosteroid eye drops or tacrolimus 0.03% eye ointment without interruption of dupilumab (96, 109).

**Tralokinumab and lebrikizumab** (Table 1) (96, 97, 110) are both approved by the European Medicines Agency (EMA) for moderate-to-severe AD in adolescents aged 12–17 years and adults. No laboratory tests are required for monitoring.

**Tralokinumab**. Three phase III studies have documented the efficacy and safety of tralokinumab in adults (111, 112). In a phase IIb trial in adults, the frequency of IGA (0, 1) responses was higher with increasing doses (45 < 150 < 300 mg subcutaneously twice monthly) (113, 114). Approval in adolescents is based on a 52-week phase III study (115, 116). The most common adverse events were upper respiratory tract infections and conjunctivitis, which developed in 7.5% of patients (113, 117). Reduction of both conjunctival goblet cells and mucin production may provoke conjunctivitis associated with IL-13 antagonists (118).

**Lebrikizumab** showed in two phase II studies in adults a significant improvement in EASI-50 at weeks 12 and 16 (111, 119). In two 16-week phase III trials enrolling adolescents and adults (120), IGA (0, 1) and EASI-75 were reached more frequently by patients in the lebrikizumab group than in the placebo group. Efficacy and safety continued after lebrikizumab withdrawal through week 52 (121). A phase III trial (122) found that TCSs did not improve the efficacy of lebrikizumab. Conjunctivitis is a common adverse event.

## Novel biologics

Several anti-T2 cytokine biologics that inhibit interleukins other than IL-4 and IL-13 (Table 1) are in clinical development (9), but have not yet been licensed (53). Moreover, there are no pediatric trials on these novel biologics available, except for one on nemolizumab in adolescents (10).

**Nemolizumab** (123–125) (Table 1) has been used in adults with uncontrolled moderate-to-severe AD, has shown to improve pruritus, the primary outcome, sleep score, and EASI in both phase I (126) and II trials (127, 128), with efficacy up to 64 weeks (128). A dose of 30 mg subcutaneously was more effective than 10 and 90 mg every 4 weeks (127). Pruritus relief was noticeable by day 2, and sleep disturbances by day 3 (129). In a phase III trial (10), in patients aged 13 years or older, nemolizumab 60 mg/4 weeks was more effective in improving primary outcome, VAS pruritus score, and EASI and DLQI than placebo. Tolerance was good.

Fezakinumab (anti-IL-22), GBR830 (anti-OX40), itepekimab (anti-IL-33) mepolizumab (anti-IL-5), omalizumab (anti-IgE) ustekinumab (anti-IL-12/23p40), risankizumab (anti-IL-23p19), rocatinlimab (anti-OX40), secukinumab (anti-IL-17A), and tezepelumab (thymic stromal lymphopoietin) showed poor or uncertain efficacy in AD (130–147).

## Small molecules

Small molecules are chemical compounds generally <0.5 kDa that require more frequent dosing and have more off-target effects when administered systemically compared to biologics. To date, phosphodiesterase-4 (PDE-4) and Janus Kinase (JAK) inhibitors have been approved for use in pediatric AD.

## PDE-4 inhibitors (Table 1)

**Crisaborole** ointment (2% twice a day) (148) has been approved for mild-to-moderate AD by regulatory agencies in patients 3 months of age and older. It is not marketed in the European Union. Crisaborole was effective in two phase III trials (149–151). Investigator’s Static Global Assessment (ISGA) success in children aged 2–17 years with mild-to-moderate AD was achieved more frequently by crisaborole than with placebo (152). Crisaborole improves sleep disruptions (153). Crisaborole was safe, with local pain for 1–2 days being the most common adverse event. In an open-label phase IV study in infants aged 3 to < 24 months with mild-to-moderate AD, crisaborole achieved moderate ISGA success, from 20% of patients at day 8 to 30.2% at week 4, with good tolerability (154).

**Difamilast** is approved in some countries for AD in children >2 years of age and adults (155). In a 4-week phase III study in children aged 2–14 years with mild-to-moderate AD (156), difamilast ointment 0.3%, 1% twice daily, achieved IGA (0, 1) in 44.6 and 47.1%, respectively, with significant differences between both difamilast and placebo groups (18.1%). A phase III trial in infants aged >3 months to 2 years is ongoing.

**Roflumilast** cream was successful in a phase II trial in mild-to-moderate AD patients aged >12 years (157). Preliminary data from phase III trials of roflumilast cream 0.15% in adults and children aged ≥6 years are promising. A phase III trial in children aged 2–5 years is ongoing.

## Topical JAK inhibitors

**Ruxolitinib** cream 1.5% (Table 1) is an FDA-approved selective JAK1 and JAK2 inhibitor (158) with warnings for short-term use in mild-to-moderate AD with up to 20% BSA-resistance to topical agents in adults and adolescents >12 years of age. Less than 60 g/week or 100 g/2 weeks are permitted. It is not recommended with biologics, JAK inhibitors, or immunosuppressants. Two phase III trials (159) in subjects aged ≥12 years with mild-to-moderate AD showed that ruxolitinib significantly reduced itching within 12 h and skin thickening (159). Ruxolitinib cream 1.5% and 0.75% (160) twice daily for 8 weeks significantly achieved IGA (0, 1) and reduced pruritus (*p* < 0.0001). Adverse events were nasopharyngitis, burning, and pruritus at the application site, but not systemic. Plasma concentrations (161) were higher at >40% BSA, but below levels of bone marrow suppression. A phase I study in children aged 2–17 years (162) showed that one patient developed neutropenia and discontinued ruxolitinib. A phase III trial in children aged ≥2 years to < 12 years is ongoing. Overall, topical ruxolitinib is useful before systemic therapy and for proactive therapy. Trials on safety are needed.

**Tofacitinib** Oral Tofacitinib, which targets JAK1, JAK2, and JAK3 (163), has been approved for several inflammatory diseases. A phase 2a study showed that tofacitinib 2% ointment significantly improved pruritus and signs in adults with mild-to-moderate AD (164).

## Oral JAK inhibitors (Table 1)

Upadacitinib, abrocitinib, and baricitinib mainly inhibit JAK1, JAK1, and JAK1-JAK, respectively. They are approved for adolescents >12 years of age and adults with moderate-to-severe AD when drugs, including biologics, are unhelpful or contraindicated. In Europe, only adults can receive abrocitinib. Oral JAK inhibitors have been associated with cancer, major cardiovascular problems, serious infections, venous thromboembolism, and mortality (165). The EMA (166) recommended its use only when alternatives are not available in patients >65 years of age, at risk for cardiovascular disease or cancer, smokers, and with caution in patients with other risk factors for blood clots in the lungs and venous thromboembolism. Doses are reduced when possible. Patients (53) are screened for HIV, viral hepatitis B and C, and tuberculosis and receive a chest radiograph at baseline. Blood count, renal, liver, and lipid profiles, in addition to creatinine phosphokinase, are checked at baseline, at week 4, and then every 3 months.

**Upadacitinib**. Three 16-week phase III trials showed significant efficacy of oral upadacitinib 15 mg and 30 mg in adults and adolescents with moderate-to-severe AD (167, 168). One patient interrupted upadacitinib 30 mg due to anemia, two due to neutropenia, and one due to moderate acne (167, 168). One patient discontinued upadacitinib 15 mg due to acne (167). In a 16-week phase III trial in adults with moderate-to-severe AD (169), upadacitinib achieved a significantly greater reduction in pruritus NRS than dupilumab. Serious infections were more common in the upadacitinib group, while conjunctivitis was more common in the dupilumab group. There was one death in the upadacitinib group due to influenza-related pneumonia.

**Abrocitinib**. In two 12-week phase III trials in patients >12 years and older (170, 171), abrocitinib (200 mg or 100 mg) achieved significantly greater proportions of IGA (0, 1) and EASI-75 responses. In a 40-week phase III trial (172), patients >12 years of age responding to abrocitinib had less frequent flares of AD with abrocitinib 200 mg or 100 mg than with the placebo. In a 12-week phase III trial (173) in adolescents, abrocitinib 200 mg or 100 mg significantly reached IGA (0, 1) and EASI-75. Nausea, upper respiratory tract infections, acne, reduced platelet count, and headaches were reported (171, 172). In a 12-week phase III study (174), abrocitinib 100 or 200 mg and dupilumab significantly reached IGA (0, 1) and EASI-75 compared to placebo, and abrocitinib demonstrated rapid efficacy (175). However, a comparison between abrocitinib and dupilumab is lacking, and definitive conclusions cannot be drawn. A phase III study (176) showed that EASI-90 was achieved in significantly more patients with abrocitinib than with dupilumab at weeks 4 and 16. At week 2, pruritus was reduced in the abrocitinib group compared to the dupilumab group (*p* < 0.0001). Nausea, headaches, acne, or folliculitis were more common with abrocitinib than with dupilumab. Conjunctivitis occurred less frequently with abrocitinib than with dupilumab.

**Baricitinib** monotherapy or with TCSs, in 16-week phase III trials (177, 178), showed significantly greater improvement in EASI-75, IGA (0, 1), NRS, POEM, and DLQI than placebo, sustained for ≤68 weeks. Serious infections, malignancies, cardiovascular events, thromboembolic events, high blood creatine phosphokinase, and cholesterol occurred. Several trials with children are ongoing.

## Aryl hydrocarbon antagonists

**Tapinarof** (Table 1) is approved for plaque psoriasis. Tapinarof cream 1% twice daily has shown promise in adults with AD, with folliculitis being the most common adverse event (179–181). Phase III trials are currently underway in both adult and pediatric populations.

## Transient receptor potential vanilloid subfamily V member 1 (TRPV1) antagonist

**Asivatrep**. In an 8-week phase III study (182) in patients aged >12 years with mild-to-moderate AD, the primary outcome IGA (0, 1) was achieved by 36.0% in the asivatrep cream 1.0% group and 12.8% in the vehicle group (*P* < 0.001). There was a significant reduction in EASI-75, EASI-100, and VAS pruritus in the active group compared to the placebo group. Safety was good.

## Conclusion

Standard therapy for AD consists of daily emollients, allergen avoidance, education, and TCSs. In some clinical situations, TCIs and oral corticosteroids are useful. Proactive treatments with TCSs or TCIs and psychological consultations may be considered. Anti-H1 antihistamines have a poor effect on itching. New anti-inflammatory drugs have paved the way for a precision medicine strategy, but only a few are approved by regulatory agencies for use in children. In recalcitrant cases, topical PHE-4 inhibitors, both for flares and proactively, are limited by efficacy, cost, and a lack of global marketing. Ruxolitinib, a topical JAK inhibitor, has been approved and successfully used for severe pruritus in young children in some countries. In severe cases, biologics are the first option since immunosuppressive drugs can have modest results and need to be monitored for adverse events. Among the biologics, dupilumab is licensed for children aged 6 months and older, while tralokinumab and lebrikizumab are approved for adolescents >12 years of age. Oral JAK inhibitors such as upadacitinib and abrocitinib have been approved in adolescents when other drugs, including biologics, have failed or are contraindicated. The main disadvantages of oral JAK inhibitors are increased cost and laboratory monitoring due to serious adverse events. So, when biologics are not successful, unavailable, or cost-prohibitive, immunosuppressive agents can be considered. CsA seems to have a better clinical profile in children, although it is only approved for patients >16 years of age.

## Author contributions

CC, AG, GG, and GR performed the analysis of literature, drafted the manuscript, interpretation of the data, and the writing of the manuscript. All authors approved the version being submitted.
